# Supplementary material for: Patient death and nurses’ coping strategies: Perception of nurses at a tertiary referral hospital in Kenya
Source: PLoS One. 2026 Jan 6;21(1):e0339674. doi: 10.1371/journal.pone.0339674 (PMC12773807; doi:10.1371/journal.pone.0339674)
Supplement: S1 Appendix — (PDF) [file pone.0339674.s001.pdf]

S1. Appendix, Patient death and nurses' coping strategies: Focus group discussion guide

Start the session by introducing yourself and saying that we are seeking their thoughts and opinions and there are no right or wrong answers. You are welcomed but are not required to talk about personal experience.

Some of the questions will be about real issues and some will be speculative

Start by asking everyone to introduce himself or herself and briefly say how the day has been.

Death Reactions

1. What is the first thing that comes to mind when I say the word death? / How would you explain your feelings generally about death?
2. Can you tell me/us what it was like when you first encountered the death of a patient in your care?
3. How do you remember the death of your patient?
4. How did you experience the death of your patient? / How was your emotional reaction to this event?
5. Would you tell me/us what made you behave or react to death the way you did after you realized that your patient had died?
6. What is your opinion of what happened (death of a patient)?

7. Do you think that the situation that led to death was avoidable?

Coping strategies

1. What specific things did you do that helped cope following the death of your patient?
2. What do you think needs improvement to help nurses cope with death?

Choosing strategies

1. How does your character influence the choice of your coping mechanism?
2. How does your institution help nurses choose their coping strategies?

Training preparation

1. Would you tell me/us how basic training you received prepared you to handle death?
2. In your opinion, do you think that you were adequately exposed to caring for dying patient?

Recommendations for better training

1. What are some of the things, in your opinion, you think need to be included in basic training in order to prepare nurses to cope with death?

2. What are some of the things that should be excluded in the basic training of nurses on how to cope with death?

Closing

1. Do you have any other thoughts or views you would like to share?

2. What did it feel like to participate in a focus group/ Is it what you expected? (If not, what did you expect?).

Thank participants for participating in the interview and ask them to allow you to contact them in case there is a need to do so.
