# Supplementary material for: Patient death and nurses’ coping strategies: Perception of nurses at a tertiary referral hospital in Kenya
Source: PLoS One. 2026 Jan 6;21(1):e0339674. doi: 10.1371/journal.pone.0339674 (PMC12773807; doi:10.1371/journal.pone.0339674)
Supplement: S2 Appendix — (PDF) [file pone.0339674.s002.pdf]

S2 Appendix, Participants information sheet: Focus group on Patient death and coping strategies

Participants' Information sheet is used in this study to outline practical and ethical aspects of the research to ensure that necessary information is explained to participants. This participants Information sheet adopt participant Information as developed by Braun and Clarke (2013**)**

Who are the researchers and what is the purpose of the study?

We are ______________________________and ____________________. We are collecting data for a study about patient death and coping strategies used by nurses. The data will be used in understanding death and coping strategies used by nurses and anonymized extract will be used in a journal publication and conferences.

What type of data are being collected?

We are interested in collecting your demographic data, therefore a demographic data form is provided and you are asked to fill the form. Secondly, we are collecting data using a focus group discussion. A focus group is a group discussion focused on a particular topic, and in this focus group-, we are focusing on death and coping strategies used by nurses. The purpose of a focus group is to express views and opinions in real life situations. Therefore, you will be expected to talk to each other as well as the interviewers and to indicate when you agree and disagree with each other. We are interested in your views and opinions on the topic of death and coping strategies and we would like the focus group to be a lively discussion, there are no right and wrong answers to the questions you will be asked to discuss.

What will participation in the focus group involve?

This focus group will comprise 8 to 12 participants and two interviewers-who will be taking notes as well as audio recording of the session. The interview is expected to last for 1 hour and 30 minutes but the group might last up to 2 hours.

In the group, you will be asked to talk about issues relating to death and coping strategies used by nurses. The questions will relate to your perspectives and views on these topics, and not on individual practice related to death and coping strategies. However, you are welcome to share your personal experiences in the group, if it is well with you.

When is the focus group scheduled?

The group is scheduled for **_____________**. If you cannot attend the group for any reason, we will have a list of reserve participants and we will ask one of them to step into your place. Please be prompt.

What will happen on the day?

Once everyone has arrived, the focus group will introduce members present to help them know each participant. You will be given a copy of the consent form signed by the interviewer, you will also be asked to complete a demographic questionnaire. The interviewers will discuss what is going to happen in the group and you will be given an opportunity to ask any question that you might have and need clarification.

The interviewers will ask everyone to agree on some ground rules for the group (avoiding speaking over other people, being considerate of other people's feelings. Once everyone is happy for the group to begin, the moderator will switch on the recording devices and ask questions at the end of the group. The other interviewer/assistant will observe and take notes during the focus group but does not participate in any way.

The note s will help the transcriber to distinguish between all the different voices and produce a more accurate transcript.

What are the benefits of taking part?

You will not get direct monetary benefit but you will get the opportunity to participate in the research project and experience the research process from the inside, which we think is one of the most valuable things to learn about research. You will also get the opportunity to participate in lively and interesting discussion about death and coping strategies, share, and develop your views on the important professional issue.

Are there any risks involved?

There are no major risks involved but you may have minimal risk-emotional reactions-where the type of question asked may upset your or trigger reactions. If you feel depressed because of participating in this focus group, you will be referred for counseling services available at the site where the focus interview is being conducted.

Will I be identifiable?

You will not be identified in any way. The researchers make sure that the transcript is anonymized so that any identifiable information that is changed or removed will transcribe the focus group.

Can I withdraw from the research?

Once you have agreed to be part of the research, you can still withdraw at any time if you wish to withdraw. Your withdrawal will not affect your work in the institution in any way.

If you have any questions, please contact:

1. Gabriel Okombo +254 736242966

2. Peris Kiarie + 254785053567

3. Joel Seme Ambikile +255715822398
