# Supplementary material for: Patient death and nurses’ coping strategies: Perception of nurses at a tertiary referral hospital in Kenya
Source: PLoS One. 2026 Jan 6;21(1):e0339674. doi: 10.1371/journal.pone.0339674 (PMC12773807; doi:10.1371/journal.pone.0339674)
Supplement: S3 Appendix — (PDF) [file pone.0339674.s003.pdf]

S3. Appendix, Research Consent Form

I give my consent to participate in the focus group on death and Coping strategies. I understand that I am participating in the group on a voluntary basis and I am free to decline to answer any question or leave the focus group at any time, without giving reason. I also understand that I can withdraw from the research at any time before (_______), without giving a reason.

I understand that information provided is confidential and that I cannot discuss the things that other participants say in the group with other people outside the group.

I understand that the focus group will be audio-recorded and transcribed by the researchers. Only the researchers will hear the audio recording of the focus group in full.

I understand that the focus group data will be analyzed and reported in different publications. No individual will be named. All participants will be anonymous. Extracts from the focus group discussions may be quoted in other publications and presentations.

Signature of research participant	Date
--	---------------------
Signature of researcher	Date
--	---------------------

If you have any questions, please contact:

1. Gabriel Okombo +254 736,242,966
2. Peris Kiarie +254 785053567
3. Joel Seme Ambikile +255715822398
