# Supplementary material for: Patient death and nurses’ coping strategies: Perception of nurses at a tertiary referral hospital in Kenya
Source: PLoS One. 2026 Jan 6;21(1):e0339674. doi: 10.1371/journal.pone.0339674 (PMC12773807; doi:10.1371/journal.pone.0339674)
Supplement: S4 Appendix — (PDF) [file pone.0339674.s004.pdf]

S4 Appendix, Participant demographic form: Focus Group

In order to learn about the range of participants taking part in this focus group, we would be very grateful if you could answer the following questions. All information provided is anonymous and confidential

Please do not write your name but either write or circle your answer in the space provided that best applies to you.

1	Focus Group Site	Kijabe Hospital		
1	How old are you	20-25 years	26-30 years	31 and above years
2	Your Gender	Male	Female	
3	Your Religion	Christian	Muslim	Others----------
4	Your Qualification	Certificate	Diploma	Higher Diploma and above
5	Experience	Less than I year	2-3 years	Over 3 years

6	What languages do you speak (list ALL)	1. Swahili<br>2. English<br>3.
