# Supplementary material for: Patient death and nurses’ coping strategies: Perception of nurses at a tertiary referral hospital in Kenya
Source: PLoS One. 2026 Jan 6;21(1):e0339674. doi: 10.1371/journal.pone.0339674 (PMC12773807; doi:10.1371/journal.pone.0339674)
Supplement: Attachment — Submitted filename: Response to Reviewers.pdf [file pone.0339674.s006.pdf]

Re: Manuscript PONE-D-24-53361
Title: Patient death and nurses' coping strategies: Perception of nurses at a Tertiary Referral Hospital in Kenya
Journal: PLOS ONE

Dear Editors,

Thank you for your review of our manuscript titled *"Patient death and nurses' coping strategies: Perception of nurses at a Tertiary Referral Hospital in Kenya."* We greatly appreciate your constructive feedback and the opportunity to revise our work. We have carefully addressed all the editorial comments and have revised the manuscript accordingly to meet PLOS ONE's requirements.

Below, we provide a point-by-point response to the comments, with references to the specific sections and changes made in the manuscript.

Academic Editor

> When submitting your revision, we need you to address these additional requirements.
>
> 1. Please ensure that your manuscript meets PLOS ONE's style requirements, including those for file naming. The PLOS ONE style templates can be found at
>
> https://journals.plos.org/plosone/s/file?id=wjVg/PLOSOne_formatting_sample_main_body.pdf and https://journals.plos.org/plosone/s/file?id=ba62/PLOSOne_formatting_sample_title_authors_affiliations.pdf

Response: Thank you for the reminder. We have reviewed the PLOS ONE formatting style guides and have ensured that the manuscript now adheres to the journal's style requirements, including file naming conventions, title formatting, author affiliations, and manuscript structure. The updated files have been renamed and formatted accordingly.

2. Thank you for stating the following financial disclosure:

“Kijabe Hospital is a Mission hospital with a focus on underserved populations. An internal small grant of 500 USD was given to cover ethics submissions, and no author received any salary support to do this work.”

Please state what role the funders took in the study. If the funders had no role, please state: "The funders had no role in study design, data collection and analysis, decision to publish, or preparation of the manuscript."
If this statement is not correct you must amend it as needed.

Please include this amended Role of Funder statement in your cover letter; we will change the online submission form on your behalf.

Response: Thank you for your guidance. We confirm that the funders had no role in the study. We have included the following statement in our cover letter:

Role of Funder Statement:
“The funders had no role in study design, data collection and analysis, decision to publish, or preparation of the manuscript.”

3. Please amend either the title on the online submission form (via Edit Submission) or the title in the manuscript so that they are identical.

Response: Thank you for highlighting this. We have amended the title to ensure consistency between the online submission form and the manuscript.

4. We note you have included a table to which you do not refer in the text of your manuscript. Please ensure that you refer to Tables 1 to 5 in your text; if accepted, production will need this reference to link the reader to the Table.

Response: Thank you for your observation. We have revised the manuscript to ensure that all tables (Tables 1 to 5) are appropriately cited in the text at relevant points, in accordance with PLOS ONE guidelines. (Line 627, 635, 715, 736, & 768).

Reviewer #1

1. Two primary areas warrant further reflection and refinement. Firstly, certain themes and subthemes require differentiation or integration. Specifically, the subtheme “Psychological Trauma” could benefit from differentiation and could be re-labeled as “stress injuries,” employing terminology from the Stress First Aid model for Healthcare Professionals (https://www.ptsd.va.gov/disaster_events/for_providers/stress_first_aid.asp). Precision in terminology is crucial for research. While all encounters with death may technically qualify as trauma, reactions vary, necessitating careful distinction between post-traumatic stress reactions and other responses. The Stress First Aid model identifies four causes: potentially traumatic events, loss/grief, moral injury, and wear and tear, all of which are evident in the submission and carry distinct emotional implications for caregivers. The section on unexpected losses may align with the psychological trauma category, while grief, moral distress, and wear and tear could be presented separately.

Response: Thank you for your insightful feedback regarding the use of precise terminology in describing nurses’ emotional responses to patient death. In response, we have revised the subtheme title from *“Psychological Trauma”* to *“Stress Injuries”* to better reflect the spectrum of emotional and psychological responses, aligning our terminology with the Stress First Aid (SFA) model for healthcare professionals. (Line 198, 218, 220, 632 & 638). While we chose not to formally subdivide this theme into distinct subcategories, we have revised the section to explicitly acknowledge the various dimensions of stress outlined in the SFA framework—namely grief/loss, moral distress, wear and tear, and potentially traumatic events. This approach preserves thematic coherence while better capturing the complexity of nurses’ emotional burdens. Additionally, we have incorporated a brief overview of the SFA model in the literature review section to provide theoretical grounding and support for the use of this terminology. (Line 96).

2. Furthermore, the "Optimism, pessimism" category appears less substantiated than other themes.
i. Additionally, the sections on religious beliefs and cultural backgrounds could be integrated into a single theme of cultural factors, although this is a minor point. As a Western reader, further elaboration on specific cultural contexts influencing caregiver experiences would enhance the manuscript's value in informing culturally oriented support.

Response: Thank you for this observation. In response, we have strengthened the discussion of the "Optimism and Pessimism" subtheme to ensure clearer synthesis. (Line 664-680).

i. Thank you for the suggestion to combine the "religious beliefs" and "culture" subthemes. We agree these domains are closely linked. However, due to the distinct and significant role that spirituality plays in the study context—as both a belief system and a primary coping mechanism—we have chosen to retain them as separate subthemes. This allows us to honor the weight participants placed on spiritual interpretations of death, beyond cultural customs alone.

ii. Secondly, integrating existing literature into the thematic analyses would enhance the manuscript. For instance, the widely researched concept of moral distress/moral injury (Ansari, Warner, Taylor-Swanson, Wilson, Van Epps, Iacob, & Supiano, 2024), which appears in various sections, lacks reference to the substantial body of literature. Similarly, other relevant areas, such as peer support (Scott et al., 2010) and organizational approaches to well-being (Shanafelt & Noseworthy, 2017), are not referenced. Incorporating these concepts into the discussion would enrich the manuscript. This feedback also applies to certain sentences that require more thorough development (i.e. Line 650).

Response: Thank you for your valuable feedback and for highlighting areas where the integration of existing literature could strengthen the manuscript. We appreciate your suggestions and have made the following revisions:

1. Incorporation of Moral Distress/Moral Injury Literature:
We have integrated the literature on moral distress and moral injury into the thematic analysis, specifically referencing the work by Wilson et al. (2024) *(in place of Ansari et*

al. (2024) as we had difficult accessing the article) to support the discussion of the emotional and ethical challenges faced by nurses. (Line 100). This reference has been included in relevant sections, particularly where we discuss the psychological impact of patient death and the associated moral dilemmas (Line 653).

2. Peer Support and Organizational Approaches to Well-Being:
   We have also included references to studies on peer support (Scott et al., 2010) and organizational approaches to well-being (Shanafelt & Noseworthy, 2017). These references enhance the discussion on strategies to mitigate the emotional toll of patient death, including the importance of peer support systems and institutional support that foster nurse well-being. (Line 742, 786).

iii. Couple of other comments, line 161 *affirmed needs spelling correction. I prefer APA 7th editor style tables for readability.

Response: Thank you for your additional comments. In regard to spelling correction, we have reviewed the manuscript for any spelling errors to ensure accuracy. We have also revised the tables labeling to ensure they conform to PLOS ONE formatting guidelines.

Reviewer # 2

1. The introduction of the paper is rather descriptive and does not provide a more complex layer of how the current knowledge that professionals in health care may struggle with the death of patients surrounds practice. There are many theoretical frames which can support with that. Furthermore, there is no need, in the text, to be comparing degrees of engagement with patients between different professionals in order to emphasize the point that nurses are most engaged and thus affected. This is contested and depends on the context; thus, it is important to be examining it in its own merit.

Response: Thank you for your thoughtful feedback on the introduction section of the manuscript. We appreciate your insights and have made the following revisions to address your comments:

i. Complexity of the Current Knowledge: we have revised the introduction to provide a more nuanced understanding of how healthcare professionals, particularly nurses, experience patient death. Instead of simply stating that nurses are most affected, we have highlighted that the emotional burden varies depending on the role and proximity to the patient. (Line 52, 55-59).

ii. Comparing Engagement Between Professionals: In response to your suggestion, we have removed the comparison between different professionals in terms of their degree of engagement with patients. We acknowledge that this is a contested area and that such comparisons may not always be valid. Instead, the focus now remains on nurses' experiences, allowing the discussion to stand on its own merits. (Line 52, 55-59).
iii. Enhancing Theoretical Support: We have incorporated additional references to theoretical frameworks and research on topics such as moral distress, coping mechanisms, and emotional resilience. (Line 935, 951).
iv. Clarity and Flow: We have revised several sentences for clarity, ensuring a smoother transition between ideas, and more explicitly linking factors such as personal attributes, work environment, and training to nurses' emotional responses to death.
v. Training and Coping Mechanisms: We have further emphasized the importance of training in preparing nurses for the emotional challenges of death and dying.

2. Why were nurses who had received palliative care training excluded from the study? What is the reasoning?

Response: Thank you for your thoughtful question regarding the exclusion of nurses with palliative care training from the study.

The exclusion of nurses with palliative care training was intentional to focus on nurses without specialized training, under the assumption that those working in critical care settings would have developed coping mechanisms through their general nursing experience and exposure to patient death. This approach allowed the study to explore the emotional and psychological impact on nurses without formal palliative care training, but who still face similar challenges in critical care environments. The revised explanation clarifies the rationale for this decision and the study's focus on a broader perspective. (Line 149-154).

3. The methodology needs further attention and the choices made with methods require justification. Why were focus groups the best option for the purposes of this study?

Response:
Thank you for this insightful comment. We have revised the "Design, Sample and Data Collection" section to include a brief rationale for our methodological choices. Specifically, we justified the use of a qualitative phenomenological design to explore the deeply personal and emotional experiences of nurses coping with patient death. Additionally, we have clarified that focus group discussions (FGDs) were selected as the

primary method of data collection because they promote open discussion, allow participants to reflect on shared experiences, and generate rich data through group dynamics. The revised section also notes that while traditional FGDs are smaller, larger groups were managed effectively with careful moderation to ensure quality and depth of data collection. (Line 161-162).

4. Were there interviews as well? This is blurry in the text.

Response: Thank you for pointing this out. We confirm that the study used only focus group discussions (FGDs) for data collection; no individual interviews were conducted. To eliminate ambiguity, we have revised the methods section to explicitly state that FGDs were the sole method of data collection and that no one-on-one interviews were used in this study. This information has been added on the manuscript. (Line 159).

5. The methodology claims saturation of the data, yet how was saturation defined in this study and thus recognized when reached? There were only six focus group discussions. Was it possible to reach saturation?

Response: Thank you for this important observation. Saturation in this study was defined as the point at which no new themes, categories, or insights were emerging from subsequent focus group discussions. After the fifth FGD, the research team began to observe significant repetition of patterns, ideas, and experiences across participants. The sixth FGD confirmed thematic redundancy, affirming that the core issues had been adequately captured. Therefore, the decision to conclude data collection at six FGDs was based on this observed saturation, aligning with qualitative research principles. This information has been added on the manuscript. (Line 176-178).

6. The claim of a grounded theory approach is made. This is not evident in the paper or the methodology.

Response: We appreciate the editor's observation. Upon review, we acknowledge that the claim of using a grounded theory approach was inaccurate and not aligned with the study's actual methodology. The study followed a phenomenological design to explore and understand the lived experiences of nurses in coping with patient death. To analyze the qualitative data, we used inductive content analysis- an approach suitable for identifying themes in phenomenological studies where researchers derive categories from raw data without pre-imposed coding frameworks. The text has been revised to ensure consistency and remove any incorrect references to grounded theory. (Line 139-142).

7. The context was a faith-based hospital, and this needs further unpicking about foundational values of the organization and how those affect employees in that organization. Was faith a bigger part in this study?

Response: Thank you for this insightful comment. The context of the faith-based hospital indeed shaped the working environment and may have influenced how nurses experienced and coped with patient death. While the study did not set out to explore the influence of faith explicitly, we recognize that spiritual values may have informed participants' coping mechanisms and their perceptions of care and loss. To address this, we have included a brief contextual explanation of the institution's faith-based foundation and reflected on how this may have influenced participants' experiences in the discussion section. (Line 128-129;690-695).

8. The discussion is not synthesizing the findings nor does it offer inferences from the study currently. There is a need to re-work on it, not from the perspective of a summary of the findings.

Response: Thank you for the insightful comment. We acknowledge that the initial discussion section leaned toward summarizing the findings rather than synthesizing them or offering deeper inferences. In response, we have revised the discussion to more clearly interpret the findings in light of existing literature, highlight emerging patterns, and offer theoretical and practical implications for nursing practice, particularly in resource-limited, faith-based healthcare settings. The revised discussion now moves beyond summary to critically explore what the findings mean, how they connect with broader issues in end-of-life care, and what they suggest for future improvements. (Line 637-809).

9. Why are the participant characteristics reiterated in the discussion?

Response: Thank you for your valuable feedback regarding the placement of participant characteristics. We have revised the manuscript accordingly by relocating the demographic details of participants to the Methods section. (Line 207-210).

Reviewer # 3

Introduction: The introduction provides relevant literature on the topic. However, the authors acknowledge that the subject has been studied extensively, albeit not sufficiently in the specific context of this study. This point could be more strongly emphasized to justify the study's contribution.

Response: We appreciate the valuable feedback regarding the need to better emphasize the study's contribution despite the availability of existing literature on the topic. In response, we have revised the introduction to more clearly highlight the research gap, especially in the context of nurses working in low-resource settings like Kenya. Specifically, in (Lines 105-116) we have:

- Strengthened the justification for the study by emphasizing the shortage of literature on nurses' coping strategies within low- and middle-income countries, particularly Sub-Saharan Africa.
- Articulated how cultural factors and resource limitations uniquely affect coping mechanisms in the Kenyan healthcare context.
- Clarified how this study contributes to filling a contextual gap in the existing body of knowledge on death and coping strategies among nurses.
- These additions serve to underline the significance of the study and its relevance to the local and global nursing community.

1. **Methods:**
2. *L. 141:* The reported number of participants in some of the focus groups is unusually high (14?) based on standard qualitative methodology. A reference supporting this methodological choice is needed, along with an explanation of how such a large focus group was managed.

Response: Thank you for your valuable feedback.

In our initial proposal, we had planned to conduct focus group discussions (FGDs) with 6–8 participants, in line with standard qualitative research methodology. However, during implementation, one of the FGDs had 14 eligible and willing participants. All of them met the inclusion criteria and were interested in sharing their experiences. In the interest of inclusivity and acknowledging the value of their contributions, we chose not to turn any of them away.

To manage the larger group effectively, we ensured that the session duration was not shortened and that each participant had an opportunity to speak. The facilitator applied structured moderation techniques to maintain the flow of discussion and ensure balanced participation. Additionally, the topic of discussion was highly engaging and relevant to all participants, which facilitated active contribution without compromising the quality of data collected.

While traditional guidance recommends smaller FGD sizes, literature acknowledges that larger groups (up to 12 or more) can still yield rich and diverse insights if carefully facilitated (Krueger & Casey, 2009). We have now included a justification for this in the revised manuscript, along with a supporting reference. (Lines 165-173).

- What was the duration of each focus group session?
- *L. 149:* The focus group guide was not provided. Please clarify if this was included in an appendix or supplementary material.
- There is no mention of audio recordings. If recordings were not made, what does "the information was transcribed verbatim" mean?
- Clarify the researchers' backgrounds and their prior relationships, if any, with the participants.

Response: Thank you for your insightful questions. We are pleased to provide the following clarifications:

i. Duration of Focus Group Sessions:

Each focus group discussion (FGD) lasted between 1 to 2 hours, depending on the size of the group and the depth of participant engagement. This duration ensured that all participants had adequate time to contribute meaningfully to the discussion. (Line 286)

ii. Focus Group Guide

The focus group guide used to steer the discussions was included as supplementary material and labeled Appendix S1. (Line 182). It outlines the key thematic areas explored and the semi-structured questions that guided participant responses while allowing flexibility for emergent topics. It is also cited in in (Line 185)

iii. Use of Audio Recordings:

All focus group sessions were audio-recorded with prior consent from the participants. These recordings were later transcribed word for word to ensure accuracy in capturing

participants' perspectives and experiences. This clarification has been added on the manuscript. (Lines 186-187).

iv. Researchers' Backgrounds and Relationship to Participants:

The focus groups were conducted by two research investigators who were staff members at the same institution but were not assigned to the same departments as the participants. Importantly, they did not hold supervisory roles over any of the participants, which minimized power dynamics and helped foster a more open and honest environment for discussion. (Lines 188-192).

- *Grounded theory* was mentioned as the guiding framework for data analysis, but further explanation is required:
    1. The abstract refers to *inductive content analysis* rather than *grounded theory.*
    2. The rationale for choosing grounded theory in this study is unclear.
    3. Grounded theory involves open, axial, and selective coding. The manuscript does not specify whether these steps were followed in the analysis.
- *Excel software was used for data management and analysis*—please elaborate on how Excel was utilized for qualitative analysis.

Response: Thank you for your thoughtful comments regarding the use of grounded theory and the application of Excel in our qualitative data analysis. We appreciate the opportunity to clarify and strengthen the methodological transparency of our manuscript.

i. Clarification on Analytical Framework
We acknowledge the inconsistency between the abstract and the main text. While the grounded theory aims to develop theory, our study did not seek to generate theory but to understand lived experiences. Therefore, *inductive content analysis*, was employed within a phenomenological framework to identify themes emerging from participant narratives. We have revised the methodology and data analysis sections to reflect this consistent use of inductive content analysis. (Lines 139-142, 195).

ii. Rationale for Using Inductive Content Analysis
Given the exploratory nature of the study and the limited existing research on the topic, inductive content analysis was chosen to allow themes to emerge directly from participants' narratives. While some techniques overlapped with grounded

theory—such as open coding and constant comparison—our aim was not to build a theory, but to capture and organize nurses' lived experiences as authentically as possible. (Lines 195-201).

iii. Explanation of the Coding Process

Our analysis followed a structured and iterative process involving:

- Initial (open) coding of meaningful data fragments across transcripts,
- Constant comparison within and across focus groups to refine and group codes,
- Category development, reviewed collaboratively by the research team to ensure consistency and thematic saturation.

We have updated the Data Analysis section to describe this process more explicitly and aligned it with the principles of inductive content analysis. (Lines 195-201).

iv. Use of Excel in Qualitative Data Analysis

Microsoft Excel was utilized to manage and organize the coded data. Each row in our spreadsheet represented a discrete data fragment or participant quote, while columns were used to:

- Assign initial codes,
- Categorize related codes,
- Track the frequency and distribution of themes across FGDs,
- Facilitate comparisons of responses.

This structured use of Excel supported our iterative coding and collaborative team analysis. We have clarified this process in the revised manuscript for transparency. (Lines 204-205).

Results:

The findings are interesting and provide useful insights. However, there are several areas for improvement:

- Many paragraphs in the results section lack direct quotes from participants (e.g., L. 182, L. 184—these are just examples).

Response: Thank you for your feedback. We have reviewed the results section and incorporated direct quotes from participants to strengthen the findings, particularly in the following lines (228, 232, 238, 582).

- There is inconsistency in the use of quotation marks within and outside of participants' direct quotes (e.g., *L. 187*). A standardized format should be applied.

Response: Thank you for pointing out the inconsistency in the use of quotation marks. We have reviewed the manuscript and standardized the formatting to ensure that all participants' direct quotes are consistently enclosed in double quotation marks, while any commentary or paraphrasing outside the quotes remains unquoted. The issue noted in line 187 and other relevant sections has been corrected accordingly.

- Instead of labeling sub-themes as "Sub-theme one," "Sub-theme two," etc., numbering them explicitly would enhance clarity.

Response: Thank you for your valuable feedback. We have revised the labeling of sub-themes as requested, explicitly numbering them for enhanced clarity. For example, "Sub-theme one" has been changed to Sub-theme 1.

- The theme *religious beliefs and spiritual aspects* is underdeveloped despite its apparent relevance to the study's regional context. This should be expanded.

Response: Thank you for your valuable feedback. We have expanded the section on religious beliefs and spiritual aspects to provide a deeper interpretation of how religious beliefs shape nurses' perceptions and coping mechanisms in dealing with death of patients, particularly within the setting where spirituality is closely intertwined with daily life and professional practice. (Lines 298-300, 304-306).

- *L. 195:* "Sense of denial"—denial of what? Clarification is needed.

Response: Thank you for your observation. We have revised the section to clarify that the "sense of denial" refers to nurses' emotional struggle to accept the death of a patient. This denial developed when nurses were emotionally invested and was sometimes expressed by projecting blame onto doctors. (Lines 326-328).

- The sub-theme *Work environment* is comprehensive and contains valuable data on nurses' working conditions. However, some content seems tangential to the study's main research question (e.g., *L. 508*—"Nurses discussed the impact of workload on their well-being, citing difficulties in taking breaks"). A clearer link to the study's focus should be established.

Response: We appreciate your insight regarding the need to clearly align the sub-theme *Work Environment* with the study's main research question. We have revised the relevant section to explicitly link the reported experiences—such as difficulty in taking breaks — to the emotional burden and coping mechanisms nurses develop in high-pressure environments. These working conditions contribute significantly to the stress, emotional strain, and coping responses described by the participants, and are thus directly relevant to the study's focus on nurses' lived experiences and coping with patient death. (Lines 529-531, 547-548, 557-558).

- The study includes nurses from various hospital departments, including the emergency department, medical and surgical wards, intensive care unit, and maternity ward. Is there room to differentiate between the experiences of nurses from different departments? I suggest being more specific. For example, how do the experiences of nurses in high-intensity units like the emergency department and intensive care unit compare to those in medical and surgical wards? Are coping strategies influenced by the nature of the department? Addressing these distinctions could strengthen the discussion and provide deeper insights.

Response: Thank you for your valuable suggestion. We acknowledge that differentiating between the experiences of nurses across various departments would provide deeper insights into how departmental settings may influence coping strategies and emotional responses. However, since the data was not categorized by department and participants from different wards were mixed in the FGDs, we did not collect information that could allow for a direct comparison between the experiences of nurses in high-intensity units and those in general wards.

In light of this, we have acknowledged this as a limitation and highlighted the need for future research that could explore how experiences vary across different departments. (Lines 830-837).

Discussion:

- The discussion is overly long and structured around each sub-theme, rather than focusing on broader conceptual insights. A more synthesized, thematic discussion would enhance readability and impact.

Response: We appreciate your observation that the current version is overly detailed and sub-theme driven. In response, we have revised the section to present a more synthesized and thematic discussion. Word count in discussion section was originally 2959 and have reduced to 2312.

- *L. 577-584*: belong to the results section (Participants characteristics). In qualitative research, participant characteristics are sometimes included in the methods section. This depends on the journal's policy.

Response: Thank you for your valuable feedback regarding the placement of participant characteristics. We have revised the manuscript accordingly by relocating the demographic details of participants to the Methods section. (Lines 208-210)

- Some sections of the discussion repeat findings (e.g., *L. 666-693, L. 594-599*). The discussion should engage with the literature in a way that builds on the findings rather than reiterating them.

Response: Thank you for pointing out the repetition within the discussion, particularly in lines 594–599 and 666–693. We have carefully reviewed and revised these sections to eliminate redundancy and ensure that the discussion moves beyond reiteration of findings. The revised discussion now focuses more on interpreting the results in relation to existing literature, drawing out broader implications and conceptual insights. (Lines 1132-2889).

- The manuscript frequently cites studies that align with the results (e.g., *L. 608, L. 613*). However, instead of simply stating that findings are similar, the discussion should explore the implications of these comparisons. Examples:
    - *L. 609:* "These findings align with a study conducted with pediatric nurses in a large women's and child healthcare hospital in Singapore." How does this comparison deepen our understanding of the current study's findings?
    - *L. 644:* "A review article indicated that there is limited research in nursing examining the role of culture on the impact of death anxiety." How does this relate to Kenya's unique cultural context?

Response: Thank you for highlighting the need to move beyond surface-level comparisons with existing literature. We have revised the relevant sections of the discussion to not only acknowledge alignment with prior studies but also to interpret the significance of these comparisons.

In particular, we have also discussed how these similarities deepen our understanding of the emotional experiences of nurses within our context. Additionally, we have emphasized the implications of our findings in the conclusion section. (Line 819).

- *L. 774-794:* This section primarily lists relevant studies rather than engaging in a deeper discussion. The discussion should critically analyze these studies in relation to the findings of the current research.

Response: Thank you for your valuable observation. In response, we have revised lines 774–794 to move beyond listing relevant studies. The revised section now critically engages with the cited literature by comparing how various educational interventions, such as simulation and reflective activities, align or differ from the emotional needs identified in our findings. (Lines 791-795).

Study Limitations:

I suggest being more specific. For example,

- The study includes nurses from various hospital departments, including the emergency department, medical and surgical wards, intensive care unit, and maternity ward. I suggest addressing this aspect in the study's limitations as well. Should this be considered a limitation or a strength of the study?

An additional limitation should be acknowledged: the study was conducted in a single hospital, which may limit the generalizability of the findings.

Response: Thank you for your valuable feedback. We have revised the limitations section to be more specific and reflective of the study's design and context. In particular, we have acknowledged that although participants were drawn from a range of departments (e.g., emergency, ICU, maternity, medical, and surgical wards), the data were not categorized by department. This limits our ability to draw comparisons across clinical environments, which may influence emotional responses and coping strategies. We have also clarified that the single-site nature of the study may affect transferability and discussed how future research can build on these aspects. (lines 823-840).

References:

The reference list requires careful proofreading. For example, citations *4, 6, and 28* contain formatting errors.

Response: Thank you for your observations regarding the reference list. We have carefully reviewed and updated the references to include complete bibliographic details, correct DOI formatting, journal names, and consistent punctuation. (Lines 886, 890, 906, 935,951, 962 &978).

We appreciate the insightful comments and suggestions provided by the reviewers and the editorial team. We believe the revised manuscript has been significantly improved in response to the feedback. We are grateful for the opportunity to resubmit and hope that the revised version meets the requirements for publication in *PLOS ONE*. Please do not hesitate to let us know if any further revisions are needed.

Sincerely,
Dr. Peris Kiarie
On behalf of all co-authors.
