# Supplementary material for: Patient death and nurses’ coping strategies: Perception of nurses at a tertiary referral hospital in Kenya
Source: PLoS One. 2026 Jan 6;21(1):e0339674. doi: 10.1371/journal.pone.0339674 (PMC12773807; doi:10.1371/journal.pone.0339674)
Supplement: Attachment — Submitted filename: Response_to_Reviewers_auresp_2.pdf [file pone.0339674.s008.pdf]

Re: Manuscript PONE-D-24-53361
Title: Patient death and nurses' coping strategies: Perception of nurses at a Tertiary Referral Hospital in Kenya
Journal: PLOS ONE

Dear Editors,

Thank you for your review of our manuscript titled *"Patient death and nurses' coping strategies: Perception of nurses at a Tertiary Referral Hospital in Kenya."* We greatly appreciate your constructive feedback and the opportunity to revise our work. We have carefully addressed all the editorial comments and have revised the manuscript accordingly to meet PLOS ONE's requirements.

Below, we provide a point-by-point response to the comments, with references to the specific sections and changes made in the manuscript.

Reviewer's comments

Introduction:<br><br>105 – 116:<br><br>This important paragraph needs references. For example:<br><br>"… nurses working in low- and middle-income countries (LMICs) like Kenya, often operate under resource constrained environments, with limited access to psychological support, continuing professional 108 development, or structured debriefing sessions following patient deaths" (ref).<br><br>"Kenya presents a unique context due to its cultural diversity and varying beliefs surrounding death…" (ref)

Response: Thank you for this observation. We have reviewed the reference list to ensure accuracy and completeness. In response to the comment on the following sentence:

"…nurses working in low- and middle-income countries (LMICs) like Kenya, often operate under resource-constrained environments, with limited access to psychological support, continuing professional development, or structured debriefing sessions following patient deaths…"

We have now supported this statement with a relevant reference: Afulani, P. A., Ongeri, L., Kinyua, J., Temmerman, M., Mendes, W. B., & Weiss, S. J. (Year). Psychological and

physiological stress and burnout among maternity providers in a rural county in Kenya: Individual and situational predictors. Reference No. 25. (Line: 933-935).

In response to the comment on the following sentence:

"Kenya presents a unique context due to its cultural diversity and varying beliefs surrounding death…" (ref)

We have now supported this statement with a relevant reference: Gire, J. (2014). How death imitates life: Cultural influences on conceptions of death and dying. Online Readings in Psychology and Culture, 6(2). Reference list No. 26. (Line: 936-938).

Methodology:<br><br>In my opinion, there appears to be a confusion between the content analysis approach—which typically does not involve the development of themes—and thematic analysis. The reference cited by the authors clearly explains the distinctions between these two methods. I also question the appropriateness of using content analysis within a phenomenological framework.

Response: Thank you for the valuable observation. We acknowledge the conceptual distinctions between content analysis and thematic analysis, especially regarding their philosophical underpinnings and analytic procedures. After revisiting both our methodological approach and the cited reference (Vears & Gillam, 2022), we recognize that our original phrasing may have created confusion.

Our analysis followed an inductive coding process that led to the generation of themes, consistent with thematic analysis. Therefore, we have revised the abstract and methodology sections to clearly reflect that thematic analysis using inductive coding was employed. (Line: 30-31; 194-201).

Discussion:<br><br>1. In the revised version, each theme is still followed by a summary of the findings. In my opinion, it is unnecessary, as it repeats content that has already been presented in the findings. For example, in line 630 – 635: "The theme of individual process provided a comprehensive understanding of the individual factors that characterizes/influence nurses' reactions, and emotional responses to patient death. This theme is outlined through the following sub themes: Stress injuries, Optimism and Pessimism, Religious Belief, Culture

and Self-management. It also highlights the individualized and self-directed approaches nurses adopt to navigate the emotional complexities associated with their patient deaths, particularly in choosing coping strategies “Table 2”. "

Response: Thank you for the helpful observation. We have carefully revised the discussion section and removed summaries of the findings that were previously restated under each theme. (Line: 630-631; 708;727;756-757).

2. The discussion is thorough and detailed; however, it is still organized according to themes and sub-sub-themes. Perhaps this is a matter of writing style. In any case, I believe it would have been more effective to identify a few key points and develop a deeper discussion around them, rather than addressing each finding separately.

Response: Thank you for your observation. We have integrated some of the subthemes that have related points and discussed them together. (Line: 759; 787).

Conclusion: The content is repetitive. Paragraph 1 (lines 811–818) repeats findings that have already been discussed. Paragraph 2 content is detailed in the following implications section.

- **Response:** We sincerely appreciate the reviewers’ thoughtful feedback regarding the organization and content of our conclusion section. We have carefully revised this section to address the concerns about repetition. We have removed restatements of findings that were already thoroughly discussed in the results and discussion sections. (Line: 797-807)

We appreciate the insightful comments and suggestions provided by the reviewers and the editorial team. We believe the revised manuscript has been significantly improved in response to the feedback. We are grateful for the opportunity to resubmit and hope that the revised version meets the requirements for publication in *PLOS ONE*. Please do not hesitate to let us know if any further revisions are needed.

Sincerely,
Dr. Peris Kiarie
On behalf of all co-authors.
