# Supplementary material for: Patient death and nurses’ coping strategies: Perception of nurses at a tertiary referral hospital in Kenya
Source: PLoS One. 2026 Jan 6;21(1):e0339674. doi: 10.1371/journal.pone.0339674 (PMC12773807; doi:10.1371/journal.pone.0339674)
Supplement: Attachment — Submitted filename: Response_to_Reviewers_auresp_3.pdf [file pone.0339674.s009.pdf]

**Re: Manuscript PONE-D-24-53361**
**Title: Patient death and nurses' coping strategies: Perception of nurses at a Tertiary Referral Hospital in Kenya**
**Journal: PLOS ONE**

**Dear Editors,**

Thank you for your review of our manuscript titled *"Patient death and nurses' coping strategies: Perception of nurses at a Tertiary Referral Hospital in Kenya."* We greatly appreciate your feedback and the opportunity to improve our work.

In particular, we have carefully revised the discussion section as requested, restructuring it into a single integrated narrative rather than using thematic sub-sections. This new format more effectively synthesizes our findings and situates them within the broader literature, as recommended by the reviewers.

Below, we provide the response to the comments, with references to the specific sections and changes made in the manuscript.

**Reviewer's comments**

| |
|---|
| Please effect the changes to the discussion as suggested by reviewer 3 at revision 1.<br><br>The discussion section must not be sub-divided by themes and sub-themes, but must be written as a single narrative which discusses the findings and their implications. |

**Response:** Thank you for this observation. The Discussion section has been restructured into a single, cohesive narrative. The word count has been reduced from 2,029 to 1,895 words (lines 626–779).

We appreciate the opportunity to enhance the quality of our work and to resubmit, and we hope that the revised version meets the requirements for publication in PLOS ONE. Please do not hesitate to let us know if any further revisions are required.

Sincerely,
Dr. Peris Kiarie
On behalf of all co-authors.
