# Supplementary material for: Patient death and nurses’ coping strategies: Perception of nurses at a tertiary referral hospital in Kenya
Source: PLoS One. 2026 Jan 6;21(1):e0339674. doi: 10.1371/journal.pone.0339674 (PMC12773807; doi:10.1371/journal.pone.0339674)
Supplement: Attachment — Submitted filename: letter of review.docx [file pone.0339674.s005.docx]

March 22, 2025

Dear Prof. Sofer,

Subject: Review of the Manuscript: "Patient Death and Nurses' Coping Strategies: Perception of Nurses at a Tertiary Referral Hospital in Kenya"

Dear Editors,

Thank you for the opportunity to review the manuscript entitled *"Patient Death and Nurses' Coping Strategies: Perception of Nurses at a Tertiary Referral Hospital in Kenya."* The study presents valuable insights into the experiences of 50 nurses in Kenya coping with patient death. The data is rich and well-detailed, contributing to the growing body of literature on this important topic. However, the manuscript falls short of delivering the unique perspective it promises concerning the *Sub-Saharan African* context. Below are my detailed comments:

**Introduction:**

The introduction provides relevant literature on the topic. However, the authors acknowledge that the subject has been studied extensively, albeit not sufficiently in the specific context of this study. This point could be more strongly emphasized to justify the study's contribution.

**Methods:**

- *L. 141:* The reported number of participants in some of the focus groups is unusually high (14?) based on standard qualitative methodology. A reference supporting this methodological choice is needed, along with an explanation of how such a large focus group was managed.
- What was the duration of each focus group session?
- *L. 149:* The focus group guide was not provided. Please clarify if this was included in an appendix or supplementary material.
- There is no mention of audio recordings. If recordings were not made, what does "the information was transcribed verbatim" mean?
- Clarify the researchers' backgrounds and their prior relationships, if any, with the participants.
- *Grounded theory* was mentioned as the guiding framework for data analysis, but further explanation is required:
  1. The abstract refers to *inductive content analysis* rather than *grounded theory.*
  2. The rationale for choosing grounded theory in this study is unclear.
  3. Grounded theory involves open, axial, and selective coding. The manuscript does not specify whether these steps were followed in the analysis.
- *Excel software was used for data management and analysis*—please elaborate on how Excel was utilized for qualitative analysis.

**Results:**

The findings are interesting and provide useful insights. However, there are several areas for improvement:

- Many paragraphs in the results section lack direct quotes from participants (e.g., L. 182, L. 184—these are just examples).
- There is inconsistency in the use of quotation marks within and outside of participants' direct quotes (e.g., *L. 187*). A standardized format should be applied.
- Instead of labeling sub-themes as "Sub-theme one," "Sub-theme two," etc., numbering them explicitly would enhance clarity.
- The theme *Religious beliefs and spiritual aspects* is underdeveloped despite its apparent relevance to the study’s regional context. This should be expanded.
- *L. 195:* "Sense of denial"—denial of what? Clarification is needed.
- The sub-theme *Work environment* is comprehensive and contains valuable data on nurses' working conditions. However, some content seems tangential to the study’s main research question (e.g., *L. 508*—"Nurses discussed the impact of workload on their well-being, citing difficulties in taking breaks"). A clearer link to the study’s focus should be established.
- The study includes nurses from various hospital departments, including the emergency department, medical and surgical wards, intensive care unit, and maternity ward. Is there room to differentiate between the experiences of nurses from different departments? I suggest being more specific. For example, how do the experiences of nurses in high-intensity units like the emergency department and intensive care unit compare to those in medical and surgical wards? Are coping strategies influenced by the nature of the department? Addressing these distinctions could strengthen the discussion and provide deeper insights.

**Discussion:**

- The discussion is overly long and structured around each sub-theme, rather than focusing on broader conceptual insights. A more synthesized, thematic discussion would enhance readability and impact.
- *L. 577-584*: belong to the results section (Participants characteristics). In qualitative research, participant characteristics are sometimes included in the methods section. This depends on the journal's policy.
- Some sections of the discussion repeat findings (e.g.,  *L. 666-693, L. 594-599*). The discussion should engage with the literature in a way that builds on the findings rather than reiterating them.
- The manuscript frequently cites studies that align with the results (e.g., *L. 608, L. 613*). However, instead of simply stating that findings are similar, the discussion should explore the implications of these comparisons. Examples:
  - *L. 609:* "These findings align with a study conducted with pediatric nurses in a large women’s and child healthcare hospital in Singapore." How does this comparison deepen our understanding of the current study's findings?
  - *L. 644:* "A review article indicated that there is limited research in nursing examining the role of culture on the impact of death anxiety." How does this relate to Kenya's unique cultural context?
- *L. 774-794:* This section primarily lists relevant studies rather than engaging in a deeper discussion. The discussion should critically analyze these studies in relation to the findings of the current research.

**Study Limitations:**

I suggest being more specific. For example,

- The study includes nurses from various hospital departments, including the emergency department, medical and surgical wards, intensive care unit, and maternity ward. I suggest addressing this aspect in the study's limitations as well. Should this be considered a limitation or a strength of the study?
- An additional limitation should be acknowledged: the study was conducted in a single hospital, which may limit the generalizability of the findings.

**References:**

- The reference list requires careful proofreading. For example, citations *4, 6, and 28* contain formatting errors.

**Final Recommendation:**

The study presents important findings, but substantial revisions are needed before publication. Specifically, the methodology should be clarified, the results should be better integrated into the discussion, and the manuscript should provide a more conceptually driven interpretation of the findings. I appreciate the opportunity to review this work.

Best regards,

Adi Finkelstein,

Jerusalem College of Technology, Jerusalem Israel
