# Supplementary material for: Patient death and nurses’ coping strategies: Perception of nurses at a tertiary referral hospital in Kenya
Source: PLoS One. 2026 Jan 6;21(1):e0339674. doi: 10.1371/journal.pone.0339674 (PMC12773807; doi:10.1371/journal.pone.0339674)
Supplement: Attachment — Submitted filename: review 2 for submission.docx [file pone.0339674.s007.docx]

15 June 2025

Dear Editor,
Thank you for the opportunity to review the revised manuscript. I believe the authors have addressed most of my comments successfully. However, a few issues were only partially resolved. I leave it to your discretion whether the manuscript should be returned to the authors for further revision or improvement.

With best regards.

++++++++++++

Introduction:

105 – 116:

This important paragraph needs references. For example:

"… nurses working in low- and middle-income countries (LMICs) like Kenya, often operate under resource constrained environments, with limited access to psychological support, continuing professional 108 development, or structured debriefing sessions following patient deaths" (ref).

"Kenya presents a unique context due to its cultural diversity and varying beliefs surrounding death…" (ref)

Methodology:

In my opinion, there appears to be a confusion between the content analysis approach—which typically does not involve the development of themes—and thematic analysis. The reference cited by the authors clearly explains the distinctions between these two methods. I also question the appropriateness of using content analysis within a phenomenological framework.

Discussion:

1. In the revised version, each theme is still followed by a summary of the findings. In my opinion, it is unnecessary, as it repeats content that has already been presented in the findings. For example, in line 630 – 635: "The theme of individual process provided a comprehensive understanding of the individual factors that characterizes/influence nurses' reactions, and emotional responses to patient death. This theme is outlined through the following sub themes: Stress injuries, Optimism and Pessimism, Religious Belief, Culture and Self-management. It also highlights the individualized and self-directed approaches nurses adopt to navigate the emotional complexities associated with their patient deaths, particularly in choosing coping strategies “Table 2”. "
2. The discussion is thorough and detailed; however, it is still organized according to themes and sub-sub-themes. Perhaps this is a matter of writing style. In any case, I believe it would have been more effective to identify a few key points and develop a deeper discussion around them, rather than addressing each finding separately.

Conclusion: The content is repetitive. Paragraph 1 (lines 811–818) repeats findings that have already been discussed. Paragraph 2 content is detailed in the following implications section.
